# Interest Groups as an Alternative for Medical Education in Sleep Medicine: Experience Report at a Brazilian Medical School

**DOI:** 10.3390/clockssleep4040053

**Published:** 2022-11-25

**Authors:** Vitor Luiz Selva Pinto, Guilherme El-Kadre, Henrique Lobo Ramos, Lucas Boaventura Pinto, Victor Davis Apostolakis Malfatti, Paula Araujo, Sandra Doria Xavier, Gabriel Natan Pires

**Affiliations:** 1 Santa Casa de São Paulo School of Medical Sciences, São Paulo 01221-020, Brazil; 2 Departamento de Biorregulação, Instituto de Ciências da Saúde, Universidade Federal da Bahia, Bahia 40110-100, Brazil; 3 Departamento de Psicobiologia, Universidade Federal de São Paulo, São Paulo 04724-000, Brazil

**Keywords:** interests group, sleep, sleep medicine, students, medical education

## Abstract

Sleep medicine classes and teachings are usually deficient and insufficient during undergraduate medical education. In order to circumvent the educational deficits in sleep medicine, students at a Brazilian Medical School created a sleep medicine interest group—an academic organization for teaching purposes whose administration is carried out by the undergraduate students themselves. This study aims to describe the establishment of a sleep medicine interest group, as well as to evaluate the results of its first edition on the knowledge about sleep medicine among undergraduate medical students. Classes were taken biweekly and consisted of lectures by invited professors, presentation of clinical cases, and discussion with the students. By the end of the course, both attendees and non-attendees were invited to fill out a questionnaire including an objective assessment of knowledge (15 multiple choice questions). The questionnaire was filled out by 32 participants, of which 18 were attendees and 14 were non-attendees. The average result on the final exam was significantly higher among the attendees (6.1 ± 1.2) in comparison with non-attendees (4.9 ± 1.3—*p* = 0.015). The results demonstrate that an interest group proved to be feasible as a source of complementary information to undergraduate medical students and a valid alternative to circumvent the educational deficits.

## 1. Introduction

Sleep research took its fundamental steps between the decades of 1940 and 1960, when remarkable achievements were taken, including the initial descriptions of the brain structures promoting sleep and wakefulness, the physiological definition of sleep stages, reports of sleep deprivation effects, and the first guides on the diagnosis of sleep disorders and the scoring of sleep-related physiological events. This knowledge set the foundations of sleep medicine as we know it today: a multidisciplinary field intending to understand, prevent, diagnose, and treat sleep-related complaints, symptoms, and disorders.

Since these initial accomplishments, sleep medicine has gained importance based on two main aspects: (1) the increasing prevalence of sleep disorders (including their consequences) and (2) its close relation with other medical conditions. In the first case, the prevalence of obstructive sleep apnea is estimated at around 30% [1,2], while the prevalence of insomnia is of 15% [3]. Furthermore, the prevalence of these conditions is likely to continue growing, as their main risk factors are also becoming increasingly common [4]. In the second case, sleep symptoms and complaints are associated with many clinical conditions in most medical specialties, which reinforces its multidisciplinary nature. As an example, sleep-related symptoms and complaints are observed in conditions such as depression [5], anxiety [6], cardiovascular disease [7,8], diabetes [9], and public health conditions such as a higher risk of work and motor vehicle accidents [10].

In the current scenario, it is necessary to emphasize the importance of evidence-based practice in sleep medicine. A proper implementation of evidence-based sleep medicine requires that professionals have access to specific and supported knowledge on the subject. Given the ubiquity of sleep-related clinical conditions in everyday medical practice, one would expect this knowledge to be transmitted during the period of supervised learning and training in medical school.

The lack of sleep-related knowledge provided in medical schools has been a matter of concern for a long time. The first works to have raised these issues were published in the 1990s, and the conclusions were that sleep-related information was deficient and insufficient during medical education [11,12,13,14]. Although important improvements were made recently (including the increase in the number of sleep medicine fellowships or residency programs), the actual coverage of sleep medicine in medical curricula seems to not have changed considerably in the last couple of decades [15], and the need for a better sleep medicine knowledge during medical education still remains. This seems to be valid in several levels of medical education, including during residency and among fellowship programs [16,17,18,19], but it is critical among undergraduate medical students [20,21,22,23].

In face of this drawback on medical curricula, students need to seek extracurricular knowledge in order to assure a proper qualification on sleep medicine. The establishment of interest groups dedicated to sleep medicine are an interesting way to circumvent this problem. Although their definition might vary considerably, undergraduate interest groups could be understood as academical organizations for teaching purposes whose initiative and administration are carried out by the graduate students themselves.

The current study aimed to describe the establishment of a sleep medicine interest group at the Santa Casa de São Paulo School of Medical Sciences (Faculdade de Ciências Médicas da Santa Casa de São Paulo—FCMSCSP), as well as to evaluate the results of its first edition on the knowledge about sleep medicine among undergraduate medical students.

## 2. Results

The sleep medicine interest group was created in the 2020 academic year by 3rd year medical students (GEK, HLR, LP, VDAM, and VLSP) under the advisory of professors from the departments of Physiological Sciences and Otolaryngology (GNP, PA, and SDX). The main objective of this interest group was to reduce the educational deficit of the university curriculum in this medical field through lectures and further teaching activities about sleep medicine. Classes were taken biweekly, most of it held online due to the restriction imposed by the COVID-19 pandemic, and consisted of lectures by invited professors, presentation of clinical cases, and discussion with the students. Overall descriptive information regarding the establishment of the sleep medicine interest group is disclosed in Table 1.

The program of the interest group was formulated to begin with topics related to basic and physiological aspects of sleep medicine, intending to create a physiological knowledge background for further clinical discussions. It was followed by lectures on clinical topics related to sleep medicine, exercising clinical reasoning, and focusing on clinical practice for a better assimilation of the content by the students. Furthermore, lectures regarding the legal and historic aspects of sleep medicine in Brazil were included. The list of lectures in the 2020 edition is presented in Table 2.

Considering all 15 classes held, we had more than 150 students enrolled, with an average of 70 students per class and a predominance of medical students.

By the end of the 2020 edition of the sleep medicine interest group, a questionnaire was created and made available to students who had or had not attended to fill it out in order to provide a proper comparator and assess the academic impact of the interest group. The questionnaire addressed students’ perception of the activity, previous knowledge related to sleep, and the obtained knowledge. Four sections of this questionnaire were used in this study, as disclosed in Section 4.

The questionnaire was filled out by 39 respondents. One was excluded for being a psychology undergraduate student from another university and six for being classified as partial attendees, leading to a final sample of 32 medical students. Among these, 18 (56.2%) were considered full attendees and 14 (43.8%) were non-attendees. Nine participants (28.1%) were 1st or 2nd graders, 15 (46.9%) were 3rd or 4th graders and eight (25.0%) were 5th or 6th graders. Most of the sample (*n* = 21; 65.6%) were male and had no previous experience or knowledge about sleep medicine before joining the interest group. The sample description is disclosed in Table 3.

**Figure 1:**
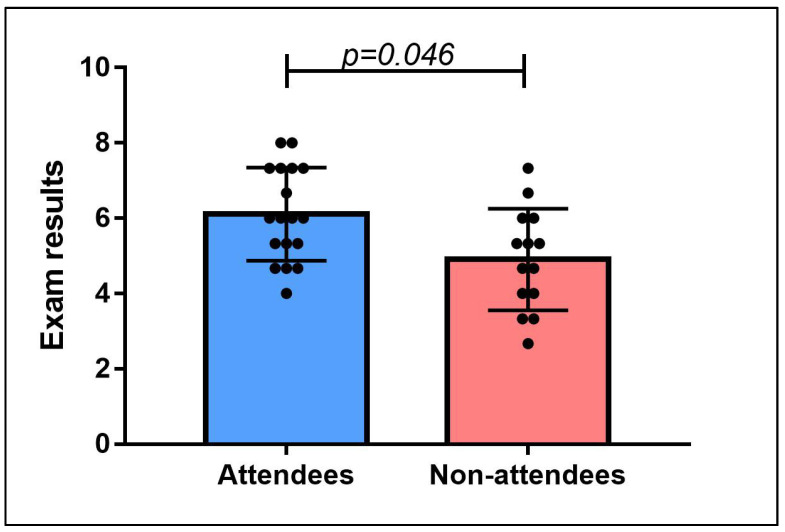
Comparison of exam results among attendees (*n* = 18) and non-attendees (*n* = 14). Data are presented as mean ± standard deviation. Color pattern for illustrative purposes only.

The participants average rating concerning the quality of activities of the interest group was 4.8 ± 0.4 (from 0 to 5). The average evaluation of the relevance of the topics was 4.7 ± 0.5 (from 0 to 5). The average likelihood to recommend a colleague to join the interest group was 9.6 ± 0.6 (from 0 to 10). The average result on the final exam was 5.6 ± 1.4 (from 0 to 10), being significantly higher among the attendees (6.1 ± 1.2) in comparison with non-attendees (4.9 ± 1.3—*p* = 0.046 – Figure 1). Gender and previous experience with sleep medicine had no statistically significant effects on the exam scores (*p* = 0.645 and *p* = 0.511, respectively).

## 3. Discussion

The current report demonstrated that sleep medicine interest groups, such as the one established at FCMSCSP, proved to be feasible as a source of complementary information to undergraduate medical students. Our results show that the specific knowledge of sleep medicine among the attendees was significantly increased, therefore, fulfilling its educational role of providing sleep medicine knowledge to the students. The actual difference in the score between the two groups was modest but still relevant. In any case, it demonstrates that even short courses such as the current one (which took 20 h in total) already lead to a better academic attainment. We wonder whether more extensive courses would promote an even better academic result.

The course was well evaluated by the students regarding their overall perception and satisfaction with the program and proposed activities. Regarding the attendance, we achieved a great engagement of both students and those who followed the project’s social networks—platforms on which various informational content was disseminated to the general public.

The deficiency in the coverage of sleep medicine in medical education is well described [15,16,17]. Many medical schools do not have a responsible department or discipline for the dissemination of sleep medicine. On the few occasions when it exists, the approach to the subject is usually fragmented into different areas of knowledge, with isolated classes in the disciplines of psychiatry, pneumology, neurology, pediatrics, and otorhinolaryngology. Short and specific medical education interventions, such as the one described in the current study, seem to be an efficient way to deal with it. Other previous studies have also demonstrated that courses and other focused didactical activities have a positive impact on the knowledge medical students have concerning sleep medicine [16,24,25]. Wappel et al. [16] demonstrated that a 2-day introductory course on sleep medicine for health professional trainees significantly improved their score on a sleep knowledge test, when compared to a baseline result. Similarly, Salas et al. [25] demonstrated that medical students who underwent an online sleep medicine learning module presented a better score on a sleep knowledge test compared to students who were not enrolled in it. Mazar et al. [24] observed that even a short educational intervention (such as a lecture) might improve medical students’ knowledge of sleep medicine.

However, in most cases, the initiative to reform the medical curriculum or to promote courses in a given field (such as in the cases disclosed above) comes from the faculty members. Differently in this case, the choice for an interest group to discuss sleep medicine in undergraduate medical courses was an organic and reactive initiative by the students. They realized this was an emergent and relevant field not properly covered in the medical curriculum and then organized themselves to promote this interest group. The fact that this is organized by the students is a key aspect of this study, as this way, the students assumed a protagonist role in their graduation, solving the limited coverage of this field and bypassing the bureaucracy of officially changing medical curricula.

Interest groups, which are loosely defined as academic organizations for teaching purposes whose administration is carried out by the graduates themselves, are a very traditional and common feature in medical schools in Brazil. As a matter of example, FCMSCSP has 44 currently active interest groups, each of them devoted to a specific medical topic. The novelty in the creation of this specific interest group relied on it being dedicated to sleep medicine, which reflects the understanding of sleep medicine as an important medical area. Similar student initiatives in other Brazilian universities are being established to try to reduce the educational deficit caused by the lack of sleep-related teaching. Along with the sleep medicine research group from FCMSCSP, described in the current report, similar initiatives were held in other Brazilian universities and medical schools in different states, such as São Paulo, Minas Gerais, and Rio Grande do Sul. Initiatives such as this tend to emerge in other universities and medical schools as they are relatively simple to organize compared to the bureaucracy needed to change the formal curriculum. In addition to reducing educational deficits, they are also an excellent personal experience for students who have a role on the board, serving as a means of preparation with responsibilities and which are valued as an extracurricular activity by teachers and medical professionals.

One factor that we considered positive in the 2020 academic year, and that will be debated in the future regarding its definitive adoption, is the use of digital platforms to carry out the lectures. This interest group was initially thought to be held exclusively in person, but online activities were enforced due to the COVID-19 pandemic and consequent social distancing norms. However, the online activities resulted in a more inclusive experience, making it more accessible and democratic—open, for example, to students from external institutions. It also allowed inviting renowned professionals from different parts of the country and the world, further favoring the dissemination of knowledge. Nevertheless, the remotely online activities imposed by the pandemic may represent some kind of limitation to the data found in the study. It is necessary to assess whether the results of an interest groups performed in a face-to-face format or in a standard classroom environment would bring similar results. In addition, it is important to cite that the attendees joined the interest group voluntarily, which might have had an impact on both the adherence to the activities and on the final results. Although not being enrolled in this specific interest group does not seem to indicate lower academic attainment or interest, as most of those in the group of non-attendants were participants in other interest groups. The outcome in a classroom environment, in which participation happens in a non-voluntary way, is still to be tested.

## 4. Materials and Methods

By the end of the 2020 edition of the sleep medicine interest group, a questionnaire was created and made available to students who had attended. A sample of students who had not attended were also invited to fill it out in order to provide a proper comparator and assess the academic impact of the interest group. The questionnaire addressed students’ perception of the activity, previous knowledge related to sleep, and the obtained knowledge. It was made available through the interest group’s social media from March 2021 to July 2021, being available to both attendees and non-attendees. Four sections of this questionnaire were used in this study, as disclosed below:

1. Identification: assessment of the student’s profile, areas of professional interest, and frequency of participation in the activities carried out
2. Previous experiences in sleep medicine: analysis of participation in other activities related to sleep medicine.
3. Interest group activities in 2020: analysis of satisfaction and perception about the activities of the sleep medicine interest group during 2020.
4. Objective assessment of knowledge in sleep medicine: to compose it, three objective questions were made for each one of the lectures composing the program of the interest group. The questions were formulated by the professors responsible for each class taught throughout the year and by the interest group organizers. Each question was composed of four multiple-choice alternatives with only one correct answer.

### 4.1. Participants and Inclusion Criteria

The only criterion for joining the interest group was being an undergraduate student in any health sciences course. Therefore, the interest group was available and open for students from courses other than medicine and for individuals coming from other universities. For the current study (rather than for the interest group enrollment), the following inclusion criteria included: being an undergraduate medical student from FCMSCSP and having completed the final exam.

The respondents were distributed in three groups according to their assiduity to classes as: full attendees (those who joined more than 75% of the classes), partial attendees (those who joined at least one but fewer than 75% of the classes), and non-attendees (those who had not attended any of the classes). Individuals classified as partial attendees were excluded from the analyses.

### 4.2. Statistical Analysis

Categorical variables were presented both as absolute frequency and percentages and were compared among groups using chi-square tests. The exam scores are presented as mean ± standard deviation and are compared between attendees and non-attendees using a three way-ANOVA (considering gender and previous experience with sleep medicine as additional independent factors). All statistical analyses were performed using JAMOVI 2.3 and the significant threshold was set at *p* < 0.05.

## 5. Conclusions

Our results demonstrate that academic initiatives held and organized by the undergraduate medical students (such as the described sleep medicine interest group) may be an effective and feasible alternative to circumvent the educational deficits in this field. By sharing this experience, we encourage the creation of other initiatives such as the one carried out in this study, showing that it is possible to disseminate knowledge about sleep medicine even outside the formal curriculum. We also hope for the future integration of these interest groups and their union to create educational events in the area.

## Figures and Tables

**Table 1:** Description of the Sleep Medicine Interest Group—2020 edition.

Item	Description
Official name	Liga Acadêmica de Medicina do Sono
Organization	Santa Casa de São Paulo School of Medical Sciences (FCMSCSP)
Creation year	2020
Activities schedule	Biweekly face-to-face meetings on Wednesdays, moving to online meetings due to the COVID-19 pandemic
Routine schedule	Usually, the following sequence of activities is followed throughout the meetings: Lecture: Initially focusing on physiology and normal sleep and, later, on sleep disorders. Clinical case presentation and discussion with the members. Questions and answers section.
Recruitment process	There was no formal recruitment. All students can participate.
Average number of members per class	70
Advisors	Gabriel Natan Pires, Paula Araujo and Sandra Doria Xavier
Cofounders	Guilherme El-Kadre, Henrique Lobo Ramos, Lucas Pinto, Victor Davis Apostolakis Malfatti, Vitor Luiz Selva Pinto
President	Vitor Luiz Selva Pinto
Email	ligamedicinasono@gmail.com
Instagram	@ligamedicinasono

**Table 2:** Program of the Sleep Medicine Interest Group—2020 edition.

Date	Topic
27 May	Normal sleep and scoring
9 June	Neurobiology of sleep
24 June	Chronobiology
1 July	Chrononutrition and nutrition related to sleep
8 July	Polysomnography
7 August	International classification of sleep disorders
19 August	Sleep pharmacology
2 September	Obstructive sleep apnea
16 September	Sleep and obesity
17 September	How to become a sleep medical doctor
23 September	Melatonin
21 October	Insomnia
28 October	Women’s sleep
11 November	Sleep and cardiovascular diseases
18 November	Pediatric sleep medicine
27 May	Normal sleep and scoring
9 June	Neurobiology of sleep
24 June	Chronobiology
1 July	Chrononutrition and nutrition related to sleep
8 July	Polysomnography

**Table 3:** Sample description.

	Total		Attendees		Non-Attendees		*p*
	*n*	%	*n*	%	*n*	%	
Sample size	32	100%	18	56.3%	14	43.8%	
Gender							
Male	21	65.6%	12	66.7%	9	64.3%	0.88
Female	11	34.4%	6	33.3%	5	35.7%	
Med School Year							
1st and 2nd	9	28.1%	4	22.2%	5	35.7%	0.70
3rd and 4th	15	46.9%	9	50.0%	6	42.9%	
5th and 6th	8	25.0%	5	27.8%	3	21.4%	
Previous experience with Sleep Medicine							
Yes	13	40.6%	7	38.9%	3	21.4%	0.29
No	19	59.4%	11	61.1%	11	78.6%	

Percentages in sample size refer to the total sample (*n* = 32), while percentages on gender, med school year, and previous experience refer to the number of respondents in the respective groups.

## Data Availability

Not applicable.
